# Supplementary material for: Understanding the Core Limitations of Second-Order Correlation-Based Functionals Through: Functional, Orbital, and Eigenvalue-Driven Analysis
Source: J Chem Theory Comput. 2025 Mar 7;21(6):2894–908. doi: 10.1021/acs.jctc.4c01376 (PMC11948335; doi:10.1021/acs.jctc.4c01376)
Supplement: Supplementary file 1 — ct4c01376_si_001.pdf [file ct4c01376_si_001.pdf]

Supporting information for "Understanding the core limitations of second-order correlation-based functionals through: functional, orbital, and eigenvalue-driven analysis"

January 20, 2025

Aditi Singh[a], Eduardo Fabiano[b,c] and Szymon Śmiga[a]
a) Institute of Physics, Faculty of Physics, Astronomy and Informatics, Nicolaus Copernicus University, Grudziadzka 5, 87-100 Toruń, Poland
b) Istituto Nanoscienze-CNR, Via per Arnesano 16, I-73100 Lecce, Italy
c) Center for Biomolecular Nanotechnologies @UNILE, Istituto Italiano di Tecnologia (IIT), Via Barsanti, 73010 Arnesano (LE), Italy

Contents:

1. **Total Energy Dataset**
2. **Binding energy Dataset**
3. **Reaction energy Dataset**

1 Total Energy Dataset

Table S1: Integrated density difference (IDD) defined as $\mathrm{IDD} = \int d\mathbf{r} |\rho^{\mathrm{CCSD(T)}}(\mathbf{r}) - \rho^{\mathrm{Method}}(\mathbf{r})|$ corresponding to various methods. The last lines report the mean absolute error (MAE) and the MAE weighted for the number of electrons (MAE/N_e).

	@HF	@SCF(GL2)[a]	@SCF(SC)	@PBE	@PBE0	@WY[HF]	@WY[MP2]	@WY[CCSD]
He	0.0105	0.0051	0.0041	0.0383	0.0210	0.0105	0.0043	0.0000
Be	0.0958	–	0.0313	0.0407	0.0390	0.0956	0.0426	0.0015
Ne	0.1021	0.1441	0.0258	0.0758	0.0251	0.1022	0.0148	0.0091
Mg	0.1264	0.0561	0.0220	0.0899	0.0399	0.1275	0.0175	0.0057
Ar	0.0634	0.0514	0.0073	0.0840	0.0592	0.0644	0.0039	0.0073
H_2	0.0282	0.0115	0.0091	0.0307	0.0155	0.0282	0.0105	0.0000
He_2	0.0210	0.0102	0.0082	0.0768	0.0421	0.0210	0.0087	0.0000
HF	0.1361	0.3228	0.0437	0.0806	0.0369	0.1366	0.0292	0.0146
CO	0.2836	0.8352	0.0814	0.1015	0.0581	0.2842	0.0582	0.0394
Cl_2	0.2581	0.2834	0.0413	0.1801	0.1237	0.2561	0.0354	0.0370
N_2	0.2303	0.6345	0.0618	0.0792	0.0524	0.2318	0.0479	0.0269
Ne_2	0.2045	0.2894	0.0518	0.1528	0.0506	0.2046	0.0293	0.0182
HCl	0.1131	0.1124	0.0228	0.1057	0.0715	0.1117	0.0280	0.0135
H_2O	0.1471	0.3997	0.0513	0.0840	0.0468	0.1478	0.0395	0.0176
NH_3	0.1330	0.2915	0.0428	0.0888	0.0508	0.1331	0.0399	0.0171
C_2H_6	0.2275	0.3171	0.0539	0.1786	0.1052	0.2260	0.0584	0.0307
MAE	0.1363	0.2510	0.0349	0.0930	0.0524	0.1363	0.0293	0.0149
MAE/N_e	0.0116	0.0187	0.0033	0.0094	0.0053	0.0116	0.0030	0.0010

[a] SCF GL2 calculations have not converged for Be. Thus, the MAE and MAE/N_e are calculated without this case

Table S2: The total energies for *ab initio* functionals (E_h) for several trial orbitals, mean error (ME), mean absolute error (MAE) are in mE_h, and mean absolute relative error (MARE), these errors were calculated with respect to CCSD(T) reference.

				@SCF		@PBE		@PBE0		WY[@HF]		WY[@MP2]		WY[@CCSD]		WY[@CCSD(T)]	
system	CCSD(T)	MP2	CCSD	GL2[a]	SEMI	GL2	SEMI	GL2	SEMI	GL2	SEMI	GL2	SEMI	GL2	SEMI	GL2	SEMI
He	-2.901132	-2.895340	-2.901132	-2.906216	-2.895440	-2.907167	-2.895357	-2.903168	-2.895407	-2.905987	-2.895339	-2.906110	-2.895380	-2.906181	-2.895390	-2.906181	-2.895390
Be	-14.659016	-14.638890	-14.658578	–	-14.639364	-14.6888261	-14.639392	-14.666119	-14.639372	-14.686111	-14.638928	-14.687817	-14.639318	-14.690098	-14.639528	-14.690205	-14.639534
Ne	-128.871168	-128.863457	-128.865425	-128.961412	-128.868320	-128.966739	-128.870145	-128.931400	-128.867982	-128.946943	-128.863711	-128.958793	-128.867988	-128.956033	-128.867112	-128.957132	-128.867456
Mg	-199.827924	-199.815445	-199.825883	-199.882751	-199.817274	-199.887942	-199.820311	-199.861383	-199.819049	-199.878108	-199.816180	-199.884293	-199.818930	-199.884065	-199.818762	-199.884321	-199.818866
Ar	-527.219879	-527.196739	-527.211413	-527.320718	-527.199601	-527.323024	-527.200160	-527.280803	-527.199298	-527.317058	-527.197783	-527.319699	-527.198877	-527.319427	-527.198797	-527.319612	-527.198845
H_2	-1.172904	-1.165258	-1.172904	-1.181588	-1.165388	-1.182043	-1.165408	-1.176119	-1.165418	-1.181088	-1.165258	-1.181484	-1.165407	-1.181740	-1.165465	-1.181740	-1.165465
He_2	-5.802265	-5.790681	-5.802265	-5.812433	-5.790881	-5.814348	-5.790713	-5.806341	-5.790815	-5.811976	-5.790680	-5.812238	-5.790762	-5.812378	-5.790782	-5.812378	-5.790782
HF	-100.404680	-100.394945	-100.396662	-100.527402	-100.400257	-100.528371	-100.403641	-100.480736	-100.400850	-100.502535	-100.395765	-100.521207	-100.401831	-100.516526	-100.400460	-100.518462	-100.401010
CO	-113.260583	-113.237272	-113.242066	-113.521317	-113.249323	-113.502859	-113.258068	-113.400786	-113.252577	-113.440148	-113.241029	-113.495931	-113.256384	-113.479528	-113.252421	-113.486945	-113.254177
H_2O	-76.394506	-76.379866	-76.385371	-76.524679	-76.384797	-76.522913	-76.387900	-76.471439	-76.385409	-76.499548	-76.380930	-76.517519	-76.386523	-76.512904	-76.385229	-76.514782	-76.385730
HCl	-460.514558	-460.487239	-460.504804	-460.625210	-460.491068	-460.628735	-460.492346	-460.579914	-460.491153	-460.619031	-460.488935	-460.624252	-460.490795	-460.623747	-460.490663	-460.624225	-460.490779
Cl_2	-919.779593	-919.730895	-919.757974	-920.014938	-919.741972	-920.020780	-919.745537	-919.917859	-919.741931	-919.992472	-919.736392	-920.010737	-919.742153	-920.007782	-919.741457	-920.010136	-919.742008
N_2	-109.479604	-109.459687	-109.459391	-109.760267	-109.470948	-109.743872	-109.478385	-109.635316	-109.473483	-109.693285	-109.464267	-109.739883	-109.477258	-109.727415	-109.474121	-109.731958	-109.475224
Ne_2	-257.742474	-257.727012	-257.730967	-257.923107	-257.736750	-257.933990	-257.740463	-257.863081	-257.736118	-257.894037	-257.727530	-257.917778	-257.736104	-257.912302	-257.734353	-257.914491	-257.735046
NH_3	-56.528936	-56.507449	-56.520253	-56.637901	-56.510850	-56.639388	-56.513430	-56.592569	-56.511740	-56.622644	-56.508323	-56.634762	-56.512186	-56.631910	-56.511387	-56.633101	-56.511694
C_2H_6	-79.766701	-79.717054	-79.751713	-79.928998	-79.722991	-79.938596	-79.727716	-79.861213	-79.725401	-79.911640	-79.719476	-79.928262	-79.724948	-79.925456	-79.724189	-79.927183	-79.724646
ME		19.92	8.70	-124.14	15.04	-118.98	12.31	-68.90	14.37	-98.54	18.46	-113.43	13.82	-110.10	14.74	-111.68	14.33
MAE		19.92	8.70	124.14	15.04	118.98	12.31	-68.90	14.37	98.54	18.46	113.43	13.82	110.10	14.74	111.68	14.33
MARE[%]		0.09	0.01	0.17	0.08	0.18	0.08	0.08	0.08	0.15	0.09	0.16	0.08	0.16	0.08	0.16	0.08

[a] SCF GL2 calculations have not converged for Be. Thus, the ME, MAE, and MARE are calculated without this case

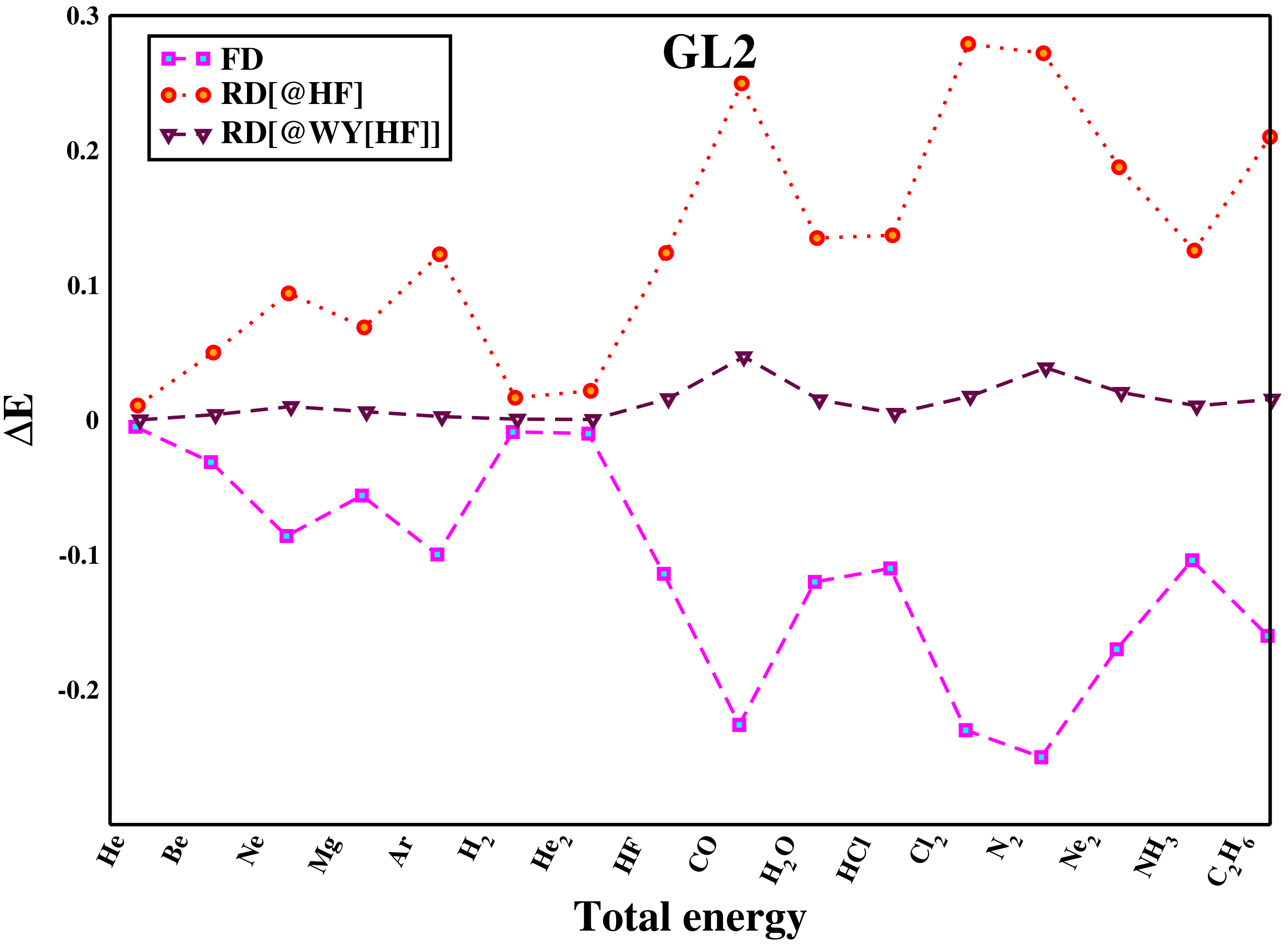

Figure S1: The FD and RD errors are calculated individually for each system in the total energy set for GL2 energy expression using @HF and @WY[HF] orbitals.

Table S3: The total energies for ISI functional (E_h) for several trial orbitals, mean error (ME), mean absolute error (MAE), are in mE_h and mean absolute relative error (MARE), these errors were calculated with respect to CCSD(T) reference.

						ISI			
system	CCSD(T)	@HF	@PBE	@PBE0	@SCF	@WY[HF]	@WY[MP2]	@WY[CCSD]	@WY[CCSD(T)]
He	-2.901132	-2.891607	-2.899811	-2.897280	-2.899632	-2.899487	-2.899567	-2.899600	-2.899600
Be	-14.659016	-14.633217	-14.669596	-14.654150	-14.670651	-14.668331	-14.669457	-14.670774	-14.670831
Ne	-128.871168	-128.835863	-128.913501	-128.888446	-128.909789	-128.900986	-128.909008	-128.907259	-128.907969
Mg	-199.827924	-199.808761	-199.873549	-199.850292	-199.869152	-199.865461	-199.870756	-199.870574	-199.870785
Ar	-527.219879	-527.183315	-527.296062	-527.259171	-527.294584	-527.291445	-527.293679	-527.293457	-527.293609
H_2	-1.172904	-1.160413	-1.170709	-1.167438	-1.170717	-1.170487	-1.170714	-1.170811	-1.170811
He_2	-5.802265	-5.783215	-5.799624	-5.794562	-5.799266	-5.798976	-5.799145	-5.799211	-5.799211
HF	-100.404680	-100.362123	-100.456200	-100.425019	-100.452522	-100.441271	-100.452727	-100.450102	-100.451217
CO	-113.260583	-113.189054	-113.365700	-113.303841	-113.360009	-113.331226	-113.363123	-113.354656	-113.358576
H_2O	-76.394506	-76.345086	-76.441840	-76.409858	-76.439323	-76.429391	-76.439670	-76.437303	-76.438288
HCl	-460.514558	-460.472908	-460.597547	-460.555614	-460.594983	-460.589984	-460.594337	-460.593932	-460.594317
Cl_2	-919.779593	-919.704232	-919.959900	-919.871434	-919.954911	-919.937136	-919.952475	-919.950070	-919.951995
N_2	-109.479604	-109.406400	-109.591567	-109.527394	-109.588605	-109.565056	-109.590550	-109.584572	-109.586794
Ne_2	-257.742474	-257.671814	-257.827364	-257.777108	-257.819783	-257.802095	-257.818171	-257.814705	-257.816115
NH_3	-56.528936	-56.474936	-56.563312	-56.534546	-56.561099	-56.554838	-56.561697	-56.560267	-56.560865
C_2H_6	-79.766701	-79.665470	-79.814362	-79.766689	-79.809098	-79.800976	-79.810695	-79.809268	-79.810138
ME		46.09	-57.17	-22.31	-54.26	-45.08	-54.37	-52.54	-53.45
MAE		46.09	57.94	25.04	55.10	46.00	55.22	53.37	54.28
MARE[%]		0.15	0.06	0.06	0.06	0.05	0.06	0.06	0.06

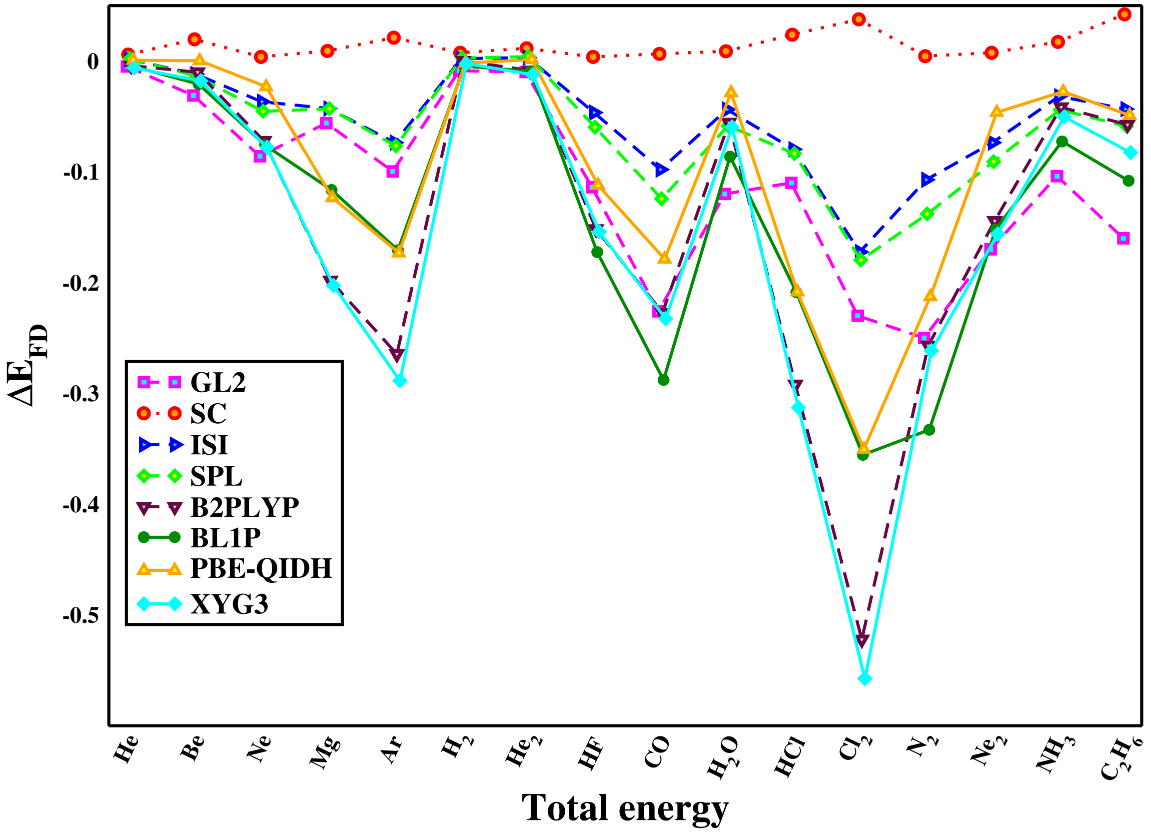

Figure S2: The individual functional driven errors ΔE_{FD} (in E_h) for total energy data set.

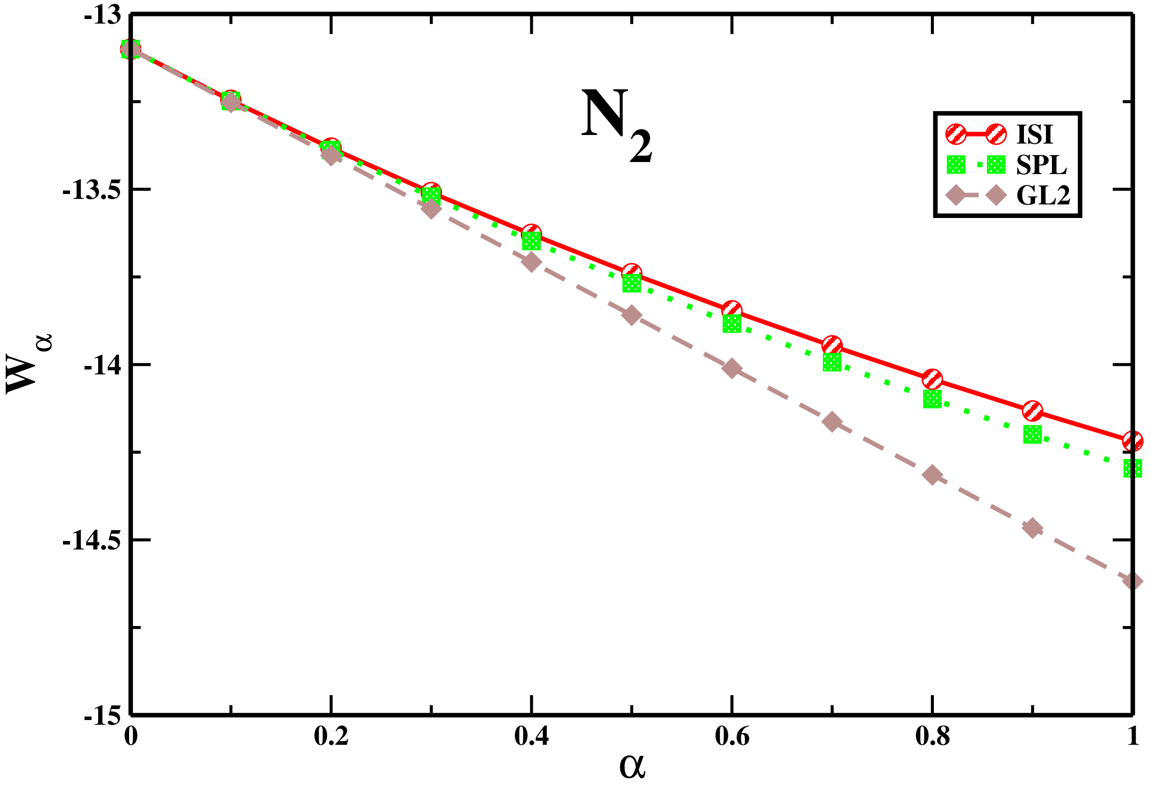

Figure S3: The AC curve W_α for N_2 molecule obtained for @WY[CCSD(T)] with uncontracted aug-cc-pVTZ basis set.

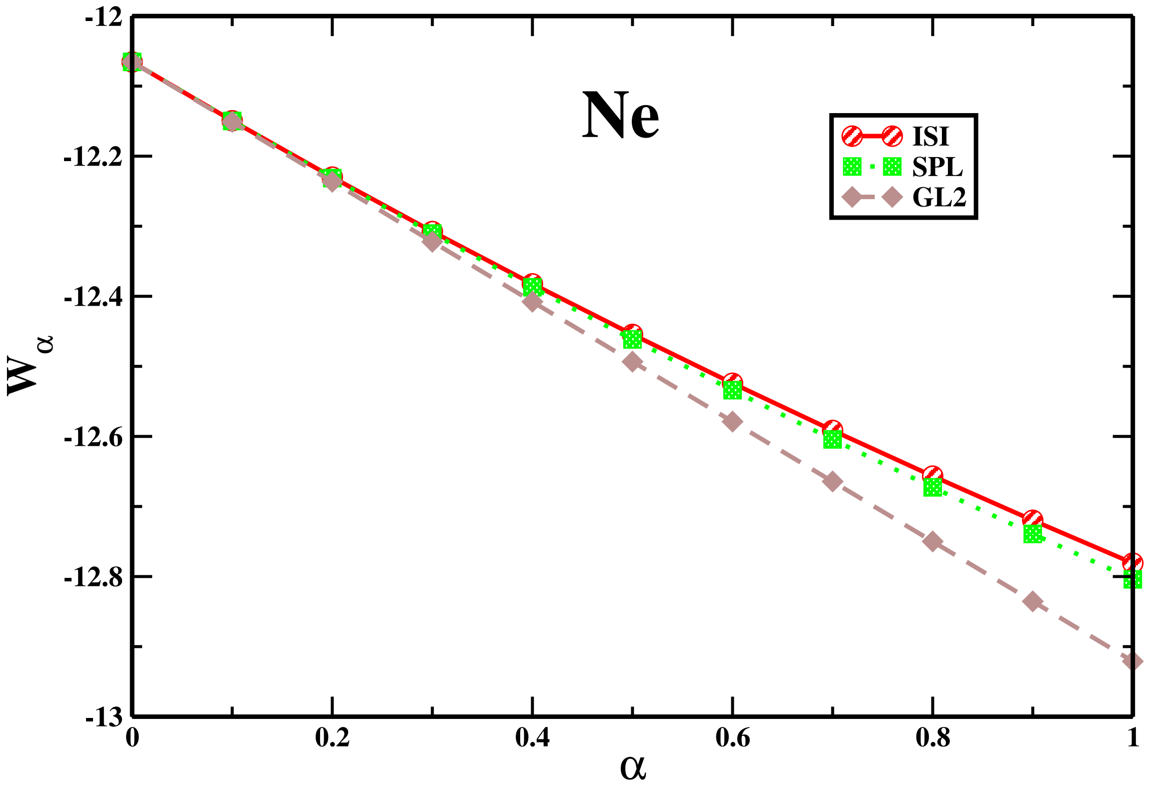

Figure S4: The AC curve W_α for Ne atom obtained for @WY[CCSD(T)] with uncontracted aug-cc-pVTZ basis set.

Table S4: The total energies for SPL functional (E_h) for several trial orbitals, the mean error (ME), mean absolute error (MAE) are in mE_h, and mean absolute relative error (MARE), these errors were calculated with respect to CCSD(T) reference.

						SPL			
system	CCSD(T)	MP2	@PBE	@PBE0	@SCF	@WY[HF]	@WY[MP2]	@WY[CCSD]	@WY[CCSD(T)]
He	-2.901132	-2.891093	-2.899358	-2.896770	-2.899158	-2.899014	-2.899093	-2.899127	-2.899127
Be	-14.659016	-14.632870	-14.671336	-14.654541	-14.672666	-14.669817	-14.671047	-14.672555	-14.672622
Ne	-128.871168	-128.838586	-128.923318	-128.895201	-128.918886	-128.908619	-128.917782	-128.915728	-128.916555
Mg	-199.827924	-199.808242	-199.873893	-199.850164	-199.869368	-199.865548	-199.870971	-199.870782	-199.871000
Ar	-527.219879	-527.183565	-527.299205	-527.261024	-527.297529	-527.294257	-527.296590	-527.296356	-527.296516
H_2	-1.172904	-1.159693	-1.170058	-1.166694	-1.170061	-1.169836	-1.170061	-1.170158	-1.170158
He_2	-5.802265	-5.782187	-5.798719	-5.793542	-5.798318	-5.798030	-5.798197	-5.798264	-5.798264
HF	-100.404680	-100.365605	-100.470938	-100.434681	-100.467154	-100.452618	-100.466315	-100.463042	-100.464414
CO	-113.260583	-113.193283	-113.394763	-113.320638	-113.391933	-113.351696	-113.390884	-113.379938	-113.384940
H_2O	-76.394506	-76.348443	-76.457974	-76.420168	-76.455607	-76.442238	-76.454848	-76.451786	-76.453044
HCl	-460.514558	-460.473226	-460.601623	-460.557946	-460.598811	-460.593510	-460.598085	-460.597653	-460.598063
Cl_2	-919.779593	-919.704660	-919.967888	-919.875774	-919.962728	-919.943697	-919.959788	-919.957237	-919.959274
N_2	-109.479604	-109.411438	-109.624645	-109.546796	-109.624150	-109.590725	-109.622753	-109.614728	-109.617680
Ne_2	-257.742474	-257.677260	-257.847034	-257.790630	-257.837995	-257.817363	-257.835723	-257.831651	-257.833295
NH_3	-56.528936	-56.477182	-56.576371	-56.542699	-56.573853	-56.565638	-56.573958	-56.572121	-56.572886
C_2H_6	-79.766701	-79.667104	-79.831480	-79.776713	-79.824880	-79.814882	-79.826265	-79.824486	-79.825567
ME		44.47	-67.67	-28.63	-64.82	-53.22	-64.15	-61.86	-62.97
MAE		44.47	68.69	31.60	65.92	54.40	65.27	62.95	64.06
MARE[%]		0.16	0.07	0.07	0.07	0.06	0.07	0.07	0.07

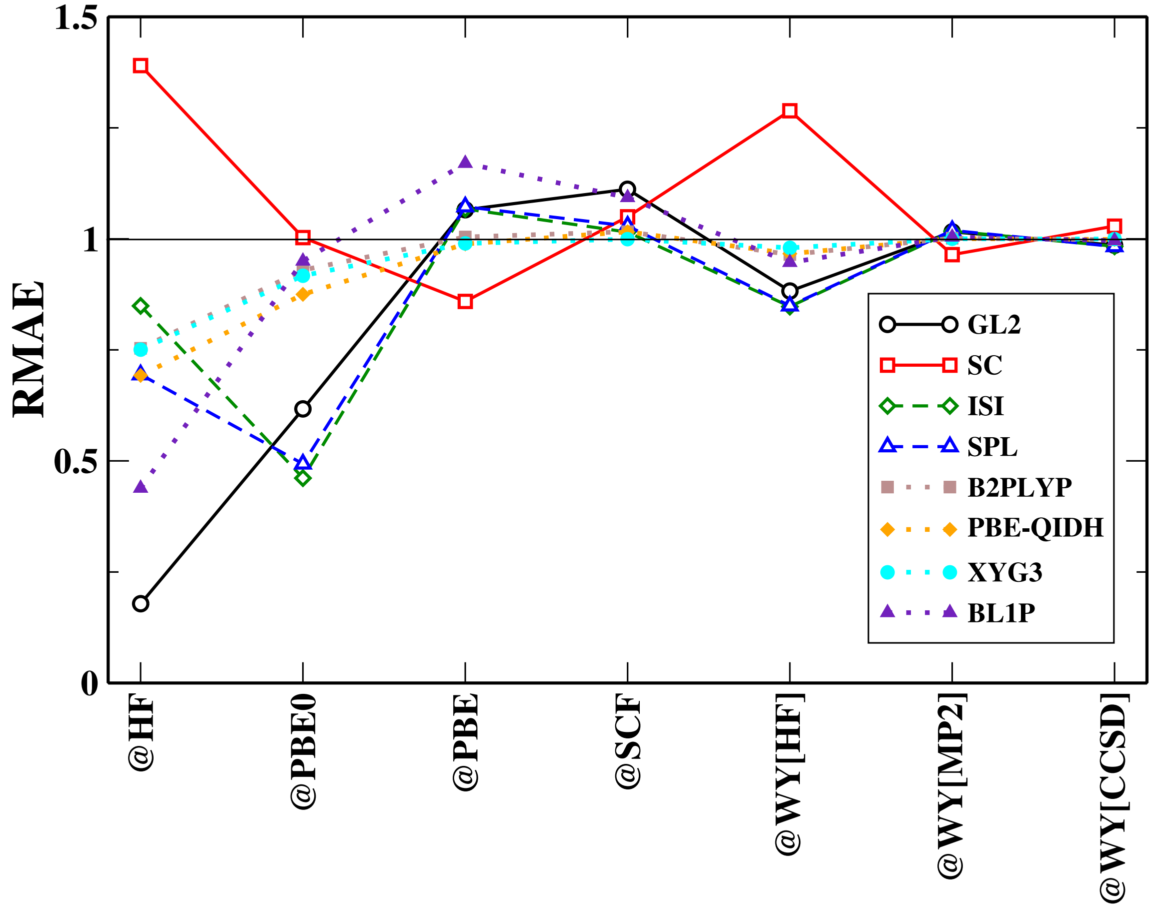

Figure S5: Relative mean absolute error (RMAE), for the total energies computed with different functionals and references. All calculations have been performed in an uncontracted aug-cc-pVTZ basis set.

Table S5: The total energies for B2PLYP functional (E_h) for several trial orbitals, mean error (ME), mean absolute error (MAE) are in mE_h, and mean absolute relative error (MARE), these errors were calculated with respect to CCSD(T) reference.

							DH			
system	CCSD(T)	@HF	@PBE	@PBE0	@SCF	@GKS	@WY[HF]	@WY[MP2]	@WY[CCSD]	@WY[CCSD(T)]
He	-2.901132	-2.902228	-2.905371	-2.904691	-2.905667	-2.903848	-2.905105	-2.905329	-2.905414	-2.905414
Be	-14.659016	-14.655432	-14.669044	-14.663331	-14.669687	-14.659862	-14.668175	-14.668908	-14.669178	-14.669191
Ne	-128.871168	-128.916420	-128.943991	-128.936973	-128.946328	-128.929849	-128.938867	-128.943684	-128.943132	-128.943379
Mg	-199.827924	-200.006242	-200.026511	-200.021082	-200.0269102	-200.016234	-200.022854	-200.026148	-200.025979	-200.026042
Ar	-527.219879	-527.450206	-527.484863	-527.475426	-527.486409	-527.466369	-527.482179	-527.484456	-527.484318	-527.484388
H_2	-1.172904	-1.168331	-1.172933	-1.171490	-1.173048	-1.170218	-1.172605	-1.172827	-1.172860	-1.172860
He_2	-5.802265	-5.804460	-5.810735	-5.809381	-5.811336	-5.807697	-5.810213	-5.810666	-5.810835	-5.810835
HF	-100.319448	-100.436532	-100.472089	-100.461995	-100.474040	-100.452701	-100.465422	-100.471644	-100.470930	-100.471271
CO	-113.126626	-113.285757	-113.355446	-113.333204	-113.358251	-113.314829	-113.339976	-113.354668	-113.352822	-113.353900
H_2O	-76.394506	-76.412371	-76.450928	-76.439425	-76.452645	-76.429333	-76.444476	-76.450490	-76.449794	-76.450136
HCl	-460.475738	-460.729679	-460.768274	-460.757471	-460.769837	-460.747434	-460.764605	-460.767754	-460.767613	-460.767727
Cl_2	-919.769257	-920.214467	-920.292594	-920.270726	-920.295597	-920.250572	-920.283206	-920.291518	-920.291027	-920.291430
N_2	-109.317341	-109.499224	-109.575576	-109.550266	-109.578146	-109.530092	-109.561733	-109.575191	-109.573450	-109.574248
Ne_2	-257.742466	-257.832841	-257.888007	-257.873994	-257.892702	-257.859736	-257.877738	-257.887383	-257.886299	-257.886782
NH_3	-56.528936	-56.535459	-56.571321	-56.560549	-56.572523	-56.551198	-56.566332	-56.570928	-56.570466	-56.570722
C_2H_6	-79.766701	-79.765373	-79.824758	-79.807688	-79.826598	-79.792406	-79.817169	-79.824224	-79.823875	-79.824250
ME		-107.48	-144.82	-133.90	-146.53	-124.19	-139.08	-144.41	-143.92	-144.20
MAE		108.67	144.82	134.08	146.53	124.53	139.12	144.42	143.92	144.21
MARE[%]		0.08	0.10	0.09	0.10	0.09	0.09	0.10	0.10	0.10

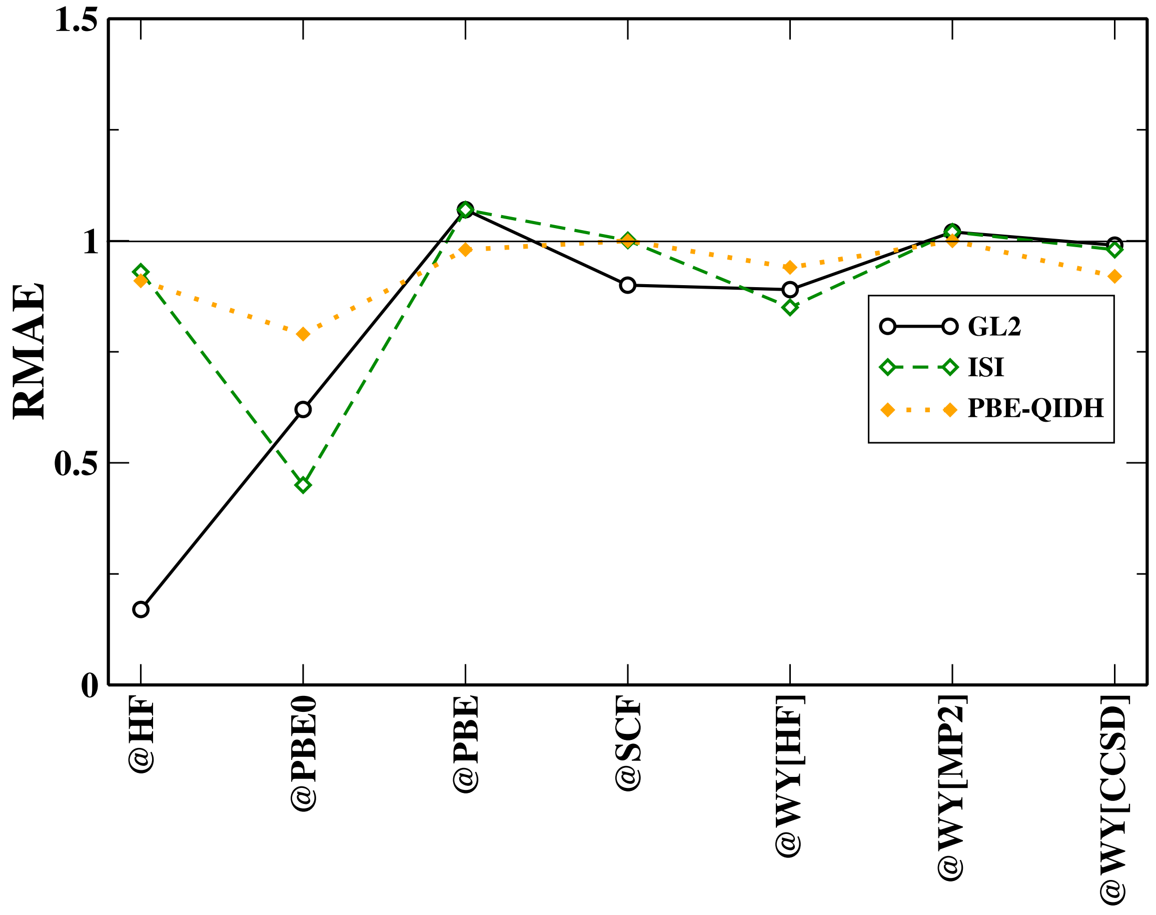

Figure S6: Relative mean absolute error (RMAE), for the total energies computed with different functionals and references. All calculations have been performed in an uncontracted aug-cc-pVQZ basis set.

Table S6: The total energies for PBE-QIDH (E_h) for several trial orbitals, mean error (ME), mean absolute error (MAE) are in mE_h, and mean absolute relative error (MARE), these errors were calculated with respect to CCSD(T) reference.

							DH			
system	CCSD(T)	@HF	@PBE	@PBE0	@SCF	@GKS	@WY[HF]	@WY[MP2]	@WY[CCSD]	@WY[CCSD(T)]
He	-2.901132	-2.896857	-2.899728	-2.899163	-2.900564	-2.897805	-2.900408	-2.900488	-2.900507	-2.900508
Be	-14.659016	-14.642367	-14.657920	-14.651231	-14.659002	-14.645274	-14.657936	-14.658604	-14.658806	-14.658805
Ne	-128.871168	-128.864755	-128.892868	-128.885645	-128.896858	-128.872935	-128.892002	-128.894378	-128.894316	-128.894390
Mg	-199.827924	-199.930334	-199.949782	-199.944395	-199.951735	-199.936091	-199.949840	-199.951333	-199.951430	-199.951418
Ar	-527.219879	-527.354430	-527.391233	-527.381043	-527.394500	-527.364887	-527.392406	-527.393164	-527.393203	-527.393171
H_2	-1.172904	-1.169418	-1.174399	-1.172877	-1.174872	-1.170656	-1.174694	-1.174829	-1.174820	-1.174821
He_2	-5.802265	-5.793715	-5.799443	-5.798321	-5.801129	-5.795611	-5.800818	-5.800983	-5.801021	-5.801021
HF	-100.319448	-100.392996	-100.430633	-100.419711	-100.433714	-100.402986	-100.428090	-100.431683	-100.431566	-100.431696
CO	-113.126626	-113.228099	-113.304898	-113.279402	-113.308624	-113.246972	-113.293527	-113.305829	-113.304595	-113.305416
H_2O	-76.394506	-76.380724	-76.422159	-76.409547	-76.424760	-76.391285	-76.419712	-76.423198	-76.423056	-76.423185
HCl	-460.475738	-460.641222	-460.682251	-460.670541	-460.685565	-460.652715	-460.682630	-460.684185	-460.68417	-460.684175
Cl_2	-919.769257	-920.033923	-920.116575	-920.092622	-920.122480	-920.057100	-920.114736	-920.119704	-920.119653	-920.119790
N_2	-109.317341	-109.444334	-109.529504	-109.500293	-109.532758	-109.464361	-109.519927	-109.530636	-109.529670	-109.530154
Ne_2	-257.742466	-257.729571	-257.785794	-257.771377	-257.793803	-257.745955	-257.784063	-257.788820	-257.788716	-257.788849
NH_3	-56.528936	-56.516620	-56.555368	-56.543595	-56.557520	-56.526599	-56.554084	-56.556637	-56.556535	-56.556613
C_2H_6	-79.766701	-79.748744	-79.813101	-79.794484	-79.816908	-79.766727	-79.810924	-79.815883	-79.815602	-79.815773
ME		-54.55	-94.40	-82.43	-97.47	-65.17	-92.53	-95.94	-95.77	-95.90
MAE		66.59	95.06	84.15	97.68	69.11	92.94	96.23	96.03	96.16
MARE[%]		0.07	0.07	0.05	0.07	0.06	0.06	0.06	0.06	0.06

Table S7: The total energies for XYG3 functional (E_h) for several trial orbitals, mean error (ME), mean absolute error (MAE) are in mE_h, and mean absolute relative error (MARE), these errors were calculated with respect to CCSD(T) reference.

							DH			
system	CCSD(T)	@HF	@PBE	@PBE0	@SCF	@GKS	@WY[HF]	@WY[MP2]	@WY[CCSD]	@WY[CCSD(T)]
He	-2.901132	-2.903791	-2.906581	-2.906048	-2.906924	-2.905976	-2.907212	-2.907314	-2.907336	-2.907337
Be	-14.659016	-14.661279	-14.676154	-14.669683	-14.676602	-14.670534	-14.676157	-14.676809	-14.677068	-14.677074
Ne	-128.871168	-128.921002	-128.947520	-128.940835	-128.950296	-128.941260	-128.946908	-128.949210	-128.949175	-128.949232
Mg	-199.827924	-200.010845	-200.029248	-200.024168	-200.029230	-200.023879	-200.029034	-200.030584	-200.030589	-200.030583
Ar	-527.219879	-527.472038	-527.506579	-527.496980	-527.507423	-527.497410	-527.507504	-527.508501	-527.508490	-527.508492
H_2	-1.172904	-1.170045	-1.174929	-1.173417	-1.175006	-1.173499	-1.175128	-1.175249	-1.175222	-1.175222
He_2	-5.802265	-5.807584	-5.813149	-5.812091	-5.813849	-5.811945	-5.814426	-5.814635	-5.814678	-5.814678
HF	-100.319448	-100.436863	-100.472377	-100.462172	-100.474588	-100.463380	-100.470237	-100.473621	-100.473527	-100.473626
CO	-113.126626	-113.287165	-113.358193	-113.334436	-113.360333	-113.337875	-113.349075	-113.359357	-113.358604	-113.359172
H_2O	-76.394506	-76.413572	-76.452933	-76.441000	-76.454638	-76.442534	-76.450628	-76.454080	-76.453916	-76.454032
HCl	-460.475738	-460.748478	-460.786700	-460.775708	-460.787628	-460.776326	-460.787102	-460.788644	-460.788619	-460.788629
Cl_2	-919.769257	-920.247311	-920.323208	-920.301123	-920.325066	-920.302637	-920.322088	-920.326634	-920.326592	-920.326672
N_2	-109.317341	-109.497965	-109.577930	-109.550343	-109.579830	-109.554561	-109.569627	-109.579200	-109.578456	-109.578868
Ne_2	-257.742466	-257.842124	-257.895161	-257.881819	-257.900746	-257.882687	-257.893926	-257.898542	-257.898493	-257.898594
NH_3	-56.528936	-56.541269	-56.578172	-56.566914	-56.579228	-56.568262	-56.576842	-56.579475	-56.579341	-56.579424
C_2H_6	-79.766701	-79.786717	-79.846242	-79.828659	-79.847854	-79.830634	-79.845225	-79.849353	-79.849251	-79.849341
ME		-115.80	-153.11	-141.88	-154.62	-143.01	-151.61	-154.74	-154.63	-154.73
MAE		116.15	153.11	141.88	154.62	143.01	151.61	154.74	154.63	154.73
MARE[%]		0.08	0.12	0.10	0.12	0.10	0.12	0.13	0.13	0.13

Table S8: The total energies for BL1P functional (E_h) for several trial orbitals, mean error (ME), mean absolute error (MAE) are in mE_h, and mean absolute relative error (MARE), these errors were calculated with respect to CCSD(T) reference.

							DH			
System	CCSD(T)	@HF	@PBE	@PBE0	@SCF[a]	@GKS	@WY[HF]	@WY[MP2]	@WY[CCSD]	@WY[CCSD(T)]
He	-2.901132	-2.898435	-2.906876	-2.905002	-2.905898	-2.899557	-2.905600	-2.905735	-2.905781	-2.905781
Be	-14.659016	-14.646528	-14.680883	-14.666455	–	-14.649574	-14.678008	-14.679071	-14.679928	-14.679962
Ne	-128.871168	-128.886271	-128.976890	-128.958040	-128.951701	-128.896055	-128.941468	-128.947628	-128.946739	-128.947139
Mg	-199.827924	-199.900910	-199.948824	-199.934517	-199.944700	-199.907457	-199.940920	-199.944252	-199.944194	-199.944255
Ar	-527.219879	-527.311429	-527.423054	-527.396987	-527.392457	-527.323157	-527.388945	-527.390712	-527.390602	-527.390674
H_2	-1.172904	-1.166726	-1.178678	-1.175083	-1.177767	-1.168136	-1.177370	-1.177610	-1.177667	-1.177667
He_2	-5.802265	-5.796873	-5.813742	-5.809999	-5.811798	-5.799117	-5.811202	-5.811482	-5.811573	-5.811574
HF	-100.319448	-100.412236	-100.517516	-100.490883	-100.497974	-100.424061	-100.483333	-100.492818	-100.491256	-100.491956
CO	-113.126626	-113.257196	-113.445244	-113.386945	-113.431614	-113.277805	-113.390089	-113.417266	-113.411861	-113.414584
H_2O	-76.394506	-76.393322	-76.501451	-76.471576	-76.486672	-76.405758	-76.472321	-76.481702	-76.480047	-76.480772
HCl	-460.475738	-460.596738	-460.713004	-460.683653	-460.686637	-460.609414	-460.681380	-460.684534	-460.684316	-460.684493
Cl_2	-919.769257	-919.948823	-920.182979	-920.122557	-920.130425	-919.974110	-920.114657	-920.124977	-920.123908	-920.124771
N_2	-109.317341	-109.475199	-109.678606	-109.613260	-109.664497	-109.497413	-109.628644	-109.652977	-109.648320	-109.650196
Ne_2	-257.742466	-257.772613	-257.953892	-257.916195	-257.903534	-257.792199	-257.883009	-257.895316	-257.893591	-257.894363
NH_3	-56.528936	-56.519627	-56.617141	-56.589456	-56.605118	-56.531165	-56.595560	-56.602104	-56.601048	-56.601540
C_2H_6	-79.766701	-79.739604	-79.897017	-79.852630	-79.878458	-79.758960	-79.866102	-79.875204	-79.874244	-79.874892
ME		-51.70	-158.78	-129.87	-148.86	-63.66	-128.96	-136.76	-135.61	-136.21
MAE		59.74	158.78	129.87	148.86	67.00	128.96	136.76	135.61	136.21
MARE [%]		0.08	0.17	0.11	0.14	0.07	0.13	0.14	0.14	0.14

[a] SCF calculations have not converged for Be. Thus, the ME, MAE, and MARE are calculated without this case

2 Binding Energy Dataset:

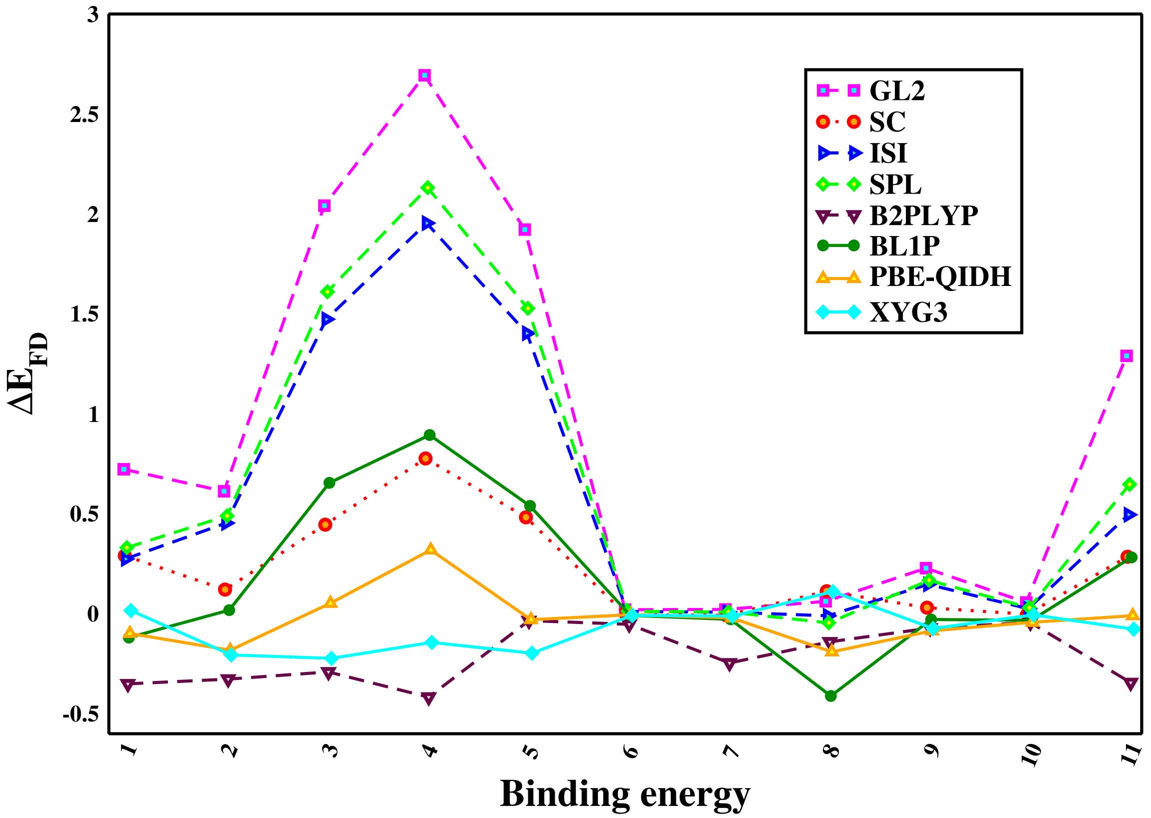

Figure S7: The individual functional driven errors ΔE_{FD} (in kcal/mol) for binding energy dataset. The numbering is described in the first column of Tab. S9.

Table S9: The binding energies for *ab initio* functionals (in kcal/mol) obtained for several trial orbitals, mean error (ME), mean absolute error (MAE), and mean absolute relative error (MARE) calculated with respect to CCSD(T) reference.

					@SCF		@PBE		@PBE0		WY[@HF]		WY[@MP2]		WY[@CCSD]		WY[@CCSD(T)]	
No.		CCSD(T)	CCSD	MP2	GL2	SC	GL2	SC	GL2	SC	GL2	SC	GL2	SC	GL2	SC	GL2	SC
1	H_2O-H_2O	5.12	4.88	5.10	5.90	5.34	5.99	5.51	5.69	5.41	5.34	5.27	5.75	5.38	5.68	5.37	5.84	5.41
2	Ar-Ar	0.28	0.21	0.34	0.81	0.36	0.78	0.38	0.61	0.37	0.84	0.40	0.89	0.40	0.88	0.40	0.89	0.40
3	H_2S-H_2S	1.89	1.57	2.16	3.98	2.33	3.94	2.37	3.23	2.31	3.71	2.29	4.01	2.36	3.84	2.32	3.93	2.34
4	H_2S-HCl	3.63	3.17	4.14	6.40	4.42	6.42	4.47	5.54	4.38	5.95	4.32	6.39	4.42	6.18	4.37	6.32	4.40
5	HCl-HCl	2.21	1.90	2.50	4.18	2.71	4.10	2.73	3.49	2.66	3.83	2.62	4.15	2.69	4.03	2.67	4.13	2.69
6	He-He	0.02	0.02	0.01	0.03	0.01	0.03	0.01	0.03	0.01	0.03	0.01	0.04	0.01	0.04	0.01	0.04	0.01
7	Ne-He	0.05	0.04	0.03	0.07	0.03	0.09	0.05	0.07	0.05	0.05	0.03	0.06	0.04	0.07	0.04	0.07	0.04
8	HF-HF	4.69	4.54	4.57	4.46	4.74	4.98	4.93	4.86	4.86	4.39	4.69	4.63	4.76	4.62	4.77	4.75	4.80
9	Ar-Ne	0.13	0.11	0.12	0.36	0.14	0.31	0.17	0.24	0.16	0.29	0.15	0.34	0.16	0.36	0.16	0.36	0.16
10	Ne-Ne	0.09	0.07	0.06	0.19	0.07	0.20	0.11	0.15	0.10	0.09	0.07	0.12	0.08	0.15	0.08	0.14	0.08
11	NH_3-NH_3	3.23	2.97	3.26	4.68	3.47	7.27	3.56	4.88	3.48	4.15	3.43	4.51	3.51	4.40	3.49	4.52	3.51
	ME		-0.17	0.09	0.89	0.21	1.16	0.27	0.68	0.22	0.67	0.18	0.87	0.23	0.81	0.21	0.88	0.23
	MAE		0.17	0.12	0.93	0.21	1.16	0.27	0.68	0.22	0.72	0.18	0.88	0.23	0.82	0.22	0.88	0.23
	MARE [%]		13.08	14.58	86.09	18.19	95.10	21.92	55.65	15.61	63.09	17.74	78.23	18.36	84.24	17.68	85.08	17.99

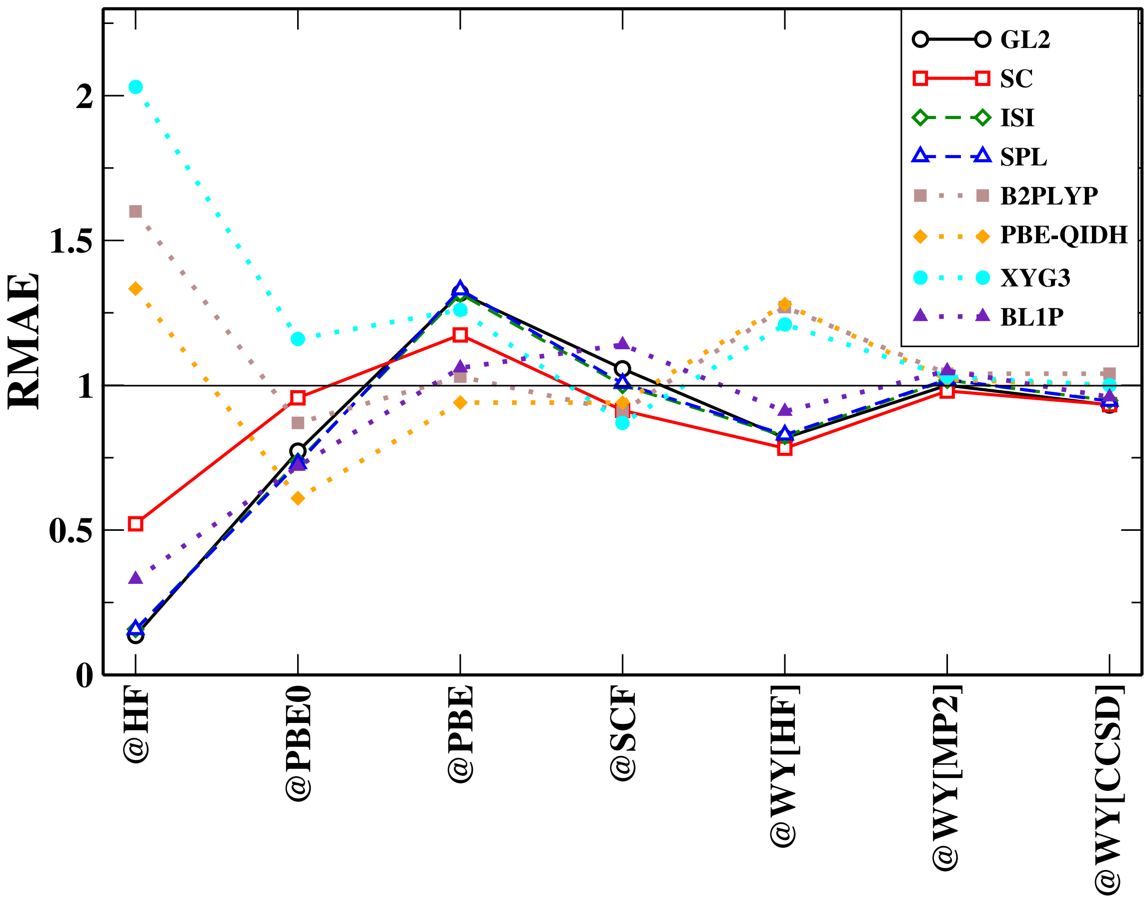

Figure S8: Relative mean absolute error (RMAE), for the binding energies computed with different functionals and references. All calculations have been performed in uncontracted aug-cc-pVTZ basis set.

Table S10: The binding energies for ISI functional (in kcal/mol) obtained for several trial orbitals, mean error (ME), mean absolute error (MAE), and mean absolute relative error (MARE) calculated with respect to CCSD(T) reference.

		ISI							
	CCSD(T)	@HF	@PBE	@PBE0	@SCF	@WY[HF]	@WY[MP2]	@WY[CCSD]	@WY[CCSD(T)]
H_2O-H_2O	5.12	4.99	5.65	5.40	5.27	5.10	5.35	5.31	5.40
Ar-Ar	0.28	0.29	0.70	0.53	0.67	0.69	0.73	0.72	0.73
H_2S-H_2S	1.89	1.98	3.62	2.88	3.35	3.19	3.43	3.29	3.36
H_2S-HCl	3.63	3.90	6.10	5.11	5.58	5.29	5.64	5.47	5.58
HCl-HCl	2.21	2.33	3.90	3.19	3.60	3.37	3.63	3.53	3.61
He-He	0.02	0.01	0.04	0.03	0.02	0.03	0.03	0.03	0.03
Ne-He	0.05	0.03	0.11	0.07	0.06	0.05	0.05	0.06	0.05
HF-HF	4.69	4.59	5.03	4.84	4.50	4.44	4.62	4.60	4.68
Ar-Ne	0.13	0.11	0.33	0.22	0.34	0.23	0.27	0.28	0.28
Ne-Ne	0.09	0.06	0.23	0.14	0.13	0.08	0.10	0.12	0.11
NH_3-NH_3	3.23	3.05	3.88	3.55	3.68	3.53	3.72	3.66	3.73
ME		0.00	0.75	0.42	0.53	0.42	0.57	0.52	0.57
MAE		0.09	0.75	0.42	0.57	0.47	0.58	0.54	0.57
MARE [%]		14.29	90.01	41.49	55.23	42.25	49.88	53.88	54.02

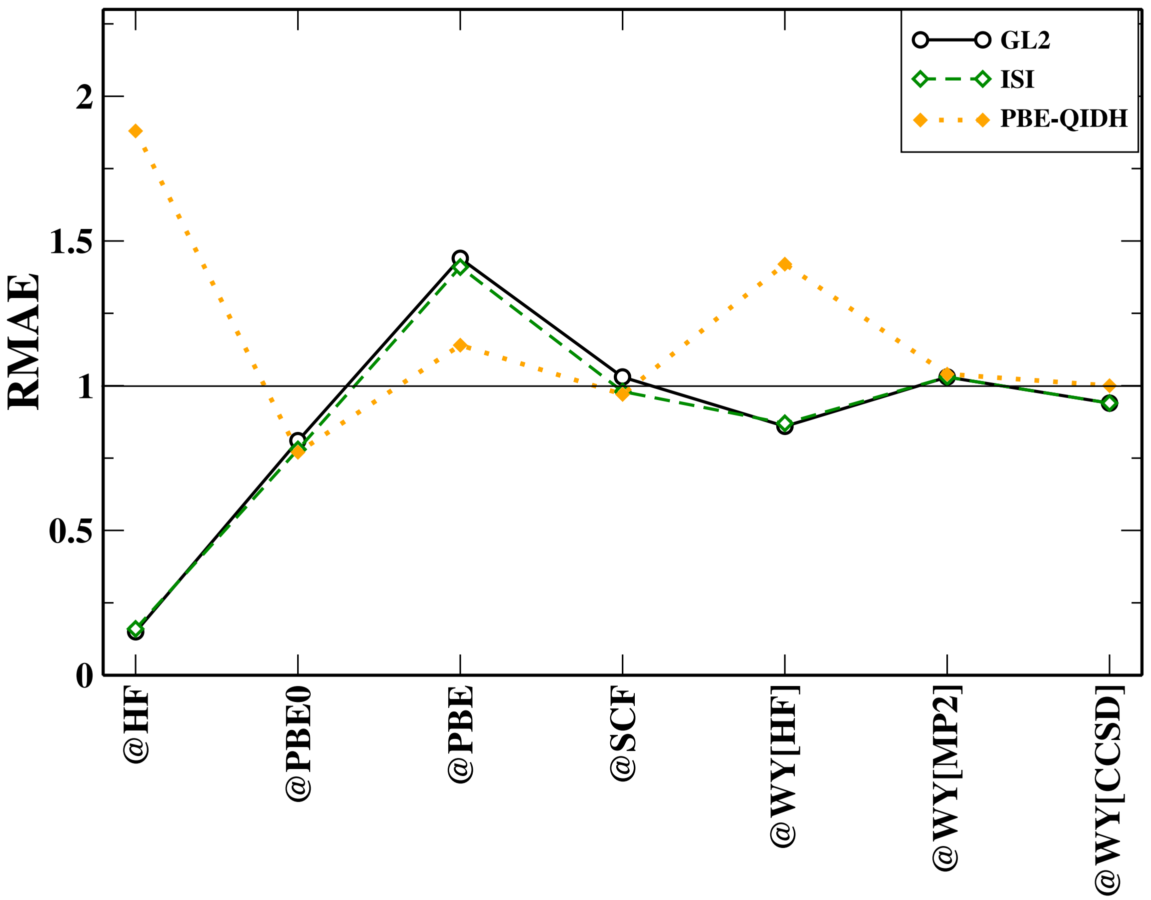

Figure S9: Relative mean absolute error (RMAE), for the binding energies computed with different functionals and references. All calculations have been performed in an uncontracted aug-cc-pVQZ basis set.

Table S11: The binding energies for SPL functionals (in kcal/mol) obtained for several trial orbitals, mean error (ME), mean absolute error (MAE), and mean absolute relative error (MARE) calculated with respect to CCSD(T) reference.

		SPL							
	CCSD(T)	@HF	@PBE	@PBE0	@SCF	@WY[HF]	@WY[MP2]	@WY[CCSD]	@WY[CCSD(T)]
H_2O-H_2O	5.12	4.98	5.79	5.44	5.31	5.10	5.39	5.34	5.45
Ar-Ar	0.28	0.30	0.74	0.55	0.70	0.72	0.77	0.75	0.77
H_2S-H_2S	1.89	2.00	3.79	2.96	3.50	3.31	3.57	3.42	3.50
H_2S-HCl	3.63	3.93	6.33	5.22	5.78	5.44	5.82	5.64	5.76
HCl-HCl	2.21	2.35	4.06	3.27	3.74	3.48	3.76	3.65	3.73
He-He	0.02	0.01	0.04	0.03	0.02	0.03	0.03	0.03	0.03
Ne-He	0.05	0.03	0.12	0.07	0.06	0.05	0.05	0.06	0.06
HF-HF	4.69	4.56	5.09	4.84	4.42	4.38	4.56	4.55	4.64
Ar-Ne	0.13	0.11	0.35	0.23	0.32	0.24	0.28	0.30	0.30
Ne-Ne	0.09	0.05	0.25	0.14	0.14	0.08	0.10	0.12	0.12
NH_3-NH_3	3.23	3.06	4.08	3.65	3.85	3.63	3.87	3.80	3.88
ME		0.01	0.85	0.46	0.59	0.47	0.62	0.58	0.63
MAE		0.10	0.85	0.46	0.64	0.53	0.65	0.60	0.64
MARE [%]		14.55	100.69	45.83	59.90	46.13	55.54	60.04	60.26

Table S12: The binding energies for B2PLYP functional (in kcal/mol) obtained for several trial orbitals, mean error (ME), mean absolute error (MAE), and mean absolute relative error (MARE) calculated with respect to CCSD(T) reference.

							DH			
	CCSD(T)	@HF	@PBE	@PBE0	@SCF	@GKS	@WY[HF]	@WY[MP2]	@WY[CCSD]	@WY[CCSD(T)]
H_2O-H_2O	5.12	4.66	4.75	4.88	4.78	4.85	4.63	4.75	4.75	4.77
Ar-Ar	0.28	-0.08	0.02	0.01	0.02	-0.02	-0.05	-0.05	-0.05	-0.05
H_2S-H_2S	1.89	1.05	1.43	1.41	1.46	1.29	1.38	1.44	1.44	1.45
H_2S-HCl	3.63	2.86	3.31	3.35	3.42	3.22	3.21	3.33	3.31	3.34
HCl-HCl	2.21	1.45	1.77	1.79	1.81	1.69	1.71	1.78	1.77	1.79
He-He	0.02	-0.02	-0.04	-0.02	-0.02	-0.02	-0.01	-0.01	-0.02	-0.02
Ne-He	0.05	-0.01	0.00	0.00	0.00	0.00	-0.01	-0.01	0.00	-0.01
HF-HF	4.69	4.46	4.40	4.60	4.46	4.61	4.32	4.42	4.42	4.44
Ar-Ne	0.13	-0.02	0.01	0.02	0.02	0.01	-0.07	-0.01	0.00	-0.01
Ne-Ne	0.09	0.00	0.02	0.03	0.03	0.02	0.00	0.01	0.02	0.02
NH_3-NH_3	3.23	2.62	2.87	2.90	2.88	2.82	2.78	2.86	2.87	2.89
ME		-0.40	-0.25	-0.21	-0.23	-0.26	-0.31	-0.26	-0.26	-0.25
MAE		0.40	0.25	0.21	0.23	0.26	0.31	0.26	0.26	0.25
MARE [%]		72.21	71.03	56.66	57.24	58.31	69.43	61.82	59.82	61.18

Table S13: The binding energies PBE-QIDH functional (kcal/mol) obtained for several trial orbitals, mean error (ME), mean absolute error (MAE), and mean absolute relative error (MARE) calculated with respect to CCSD(T) reference.

							DH			
	CCSD(T)	@HF	@PBE	@PBE0	@SCF	@GKS	@WY[HF]	@WY[MP2]	@WY[CCSD]	@WY[CCSD(T)]
H_2O-H_2O	5.12	5.02	4.98	5.14	5.02	5.15	4.89	5.03	5.01	5.02
Ar-Ar	0.28	0.09	0.18	0.17	0.15	0.12	0.11	0.10	0.10	0.10
H_2S-H_2S	1.89	1.59	1.89	1.87	1.93	1.71	1.91	1.95	1.93	1.94
H_2S-HCl	3.63	3.57	3.84	3.92	3.99	3.76	3.88	3.96	3.93	3.95
HCl-HCl	2.21	1.90	2.12	2.15	2.19	2.03	2.14	2.18	2.17	2.18
He-He	0.02	0.01	-0.01	0.01	0.01	0.02	0.02	0.02	0.01	0.01
Ne-He	0.05	0.03	0.03	0.04	0.03	0.03	0.02	0.02	0.03	0.02
HF-HF	4.69	4.64	4.44	4.66	4.50	4.75	4.40	4.50	4.49	4.50
Ar-Ne	0.13	0.05	0.07	0.08	0.07	0.07	0.01	0.04	0.06	0.05
Ne-Ne	0.09	0.04	0.04	0.05	0.05	0.05	0.04	0.04	0.05	0.04
NH_3-NH_3	3.23	3.01	3.19	3.22	3.21	3.13	3.13	3.23	3.21	3.22
ME		-0.12	-0.05	0.00	-0.02	-0.05	-0.07	-0.02	-0.03	-0.03
MAE		0.12	0.09	0.06	0.09	0.09	0.12	0.10	0.09	0.09
MARE [%]		27.30	30.95	19.07	21.05	19.24	26.97	24.49	22.29	24.44

Table S14: The binding energies for XYG3 functional (in kcal/mol) obtained for several trial orbitals, mean error (ME), mean absolute error (MAE), and mean absolute relative error (MARE) calculated with respect to CCSD(T) reference.

							DH			
	CCSD(T)	@HF	@PBE	@PBE0	@SCF	GKS	@WY[HF]	@WY[MP2]	@WY[CCSD]	@WY[CCSD(T)]
H_2O-H_2O	5.12	5.26	5.04	5.27	5.12	5.24	5.06	5.13	5.13	5.14
Ar-Ar	0.28	0.12	0.19	0.18	0.20	0.20	0.07	0.07	0.07	0.07
H_2S-H_2S	1.89	1.39	1.57	1.62	1.63	1.61	1.63	1.66	1.66	1.67
H_2S-HCl	3.63	3.24	3.30	3.47	3.46	3.45	3.43	3.48	3.48	3.48
HCl-HCl	2.21	1.83	1.92	2.01	2.01	2.01	1.97	2.00	2.01	2.01
He-He	0.02	0.01	-0.02	0.00	0.02	0.01	0.01	0.01	0.01	0.01
Ne-He	0.05	0.04	0.04	0.05	0.05	0.06	0.03	0.03	0.04	0.03
HF-HF	4.69	5.05	4.67	4.96	4.78	4.93	4.74	4.80	4.80	4.80
Ar-Ne	0.13	0.09	0.10	0.11	0.11	0.12	0.03	0.06	0.07	0.06
Ne-Ne	0.09	0.08	0.08	0.09	0.10	0.10	0.07	0.08	0.09	0.08
NH_3-NH_3	3.23	3.05	3.09	3.18	3.13	3.17	3.09	3.14	3.15	3.15
ME		-0.11	-0.12	-0.04	-0.07	-0.04	-0.11	-0.08	-0.08	-0.07
MAE		0.20	0.12	0.11	0.09	0.11	0.12	0.10	0.10	0.10
MARE [%]		22.28	28.54	16.98	9.67	15.60	24.64	21.51	20.02	21.42

Table S15: The binding energies for BL1P functional (in kcal/mol) obtained for several trial orbitals, mean error (ME), mean absolute error (MAE), and mean absolute relative error (MARE) calculated with respect to CCSD(T) reference.

							DH			
	CCSD(T)	@HF	@PBE	@PBE0	@SCF	GKS	@WY[HF]	@WY[MP2]	@WY[CCSD]	@WY[CCSD(T)]
H_2O-H_2O	5.12	4.98	5.02	5.17	4.96	5.07	4.81	4.95	4.96	5.00
Ar-Ar	0.28	0.17	0.44	0.36	0.38	0.21	0.28	0.30	0.29	0.30
H_2S-H_2S	1.89	1.70	2.55	2.34	2.58	1.84	2.43	2.56	2.52	2.55
H_2S-HCl	3.63	3.61	4.56	4.42	4.64	3.81	4.36	4.54	4.48	4.52
HCl-HCl	2.21	2.08	2.79	2.67	2.81	2.21	2.63	2.75	2.72	2.75
He-He	0.02	0.00	-0.02	0.00	0.00	0.00	0.01	0.01	0.01	0.01
Ne-He	0.05	0.02	0.03	0.03	0.026	0.03	0.02	0.01	0.03	0.02
HF-HF	4.69	4.60	4.29	4.59	4.17	4.65	4.19	4.22	4.25	4.28
Ar-Ne	0.13	0.07	0.15	0.14	0.13	0.09	0.10	0.09	0.12	0.11
Ne-Ne	0.09	0.04	0.07	0.07	0.08	0.06	0.05	0.04	0.07	0.05
NH_3-NH_3	3.23	3.03	3.50	3.43	3.54	3.13	3.34	3.49	3.47	3.51
ME		-0.09	0.19	0.17	0.18	-0.02	0.08	0.15	0.14	0.16
MAE		0.09	0.29	0.20	0.31	0.05	0.25	0.29	0.26	0.27
MARE [%]		27.26	38.00	23.79	26.17	20.42	23.35	28.21	20.22	24.89

3 Reaction Energy Dataset:

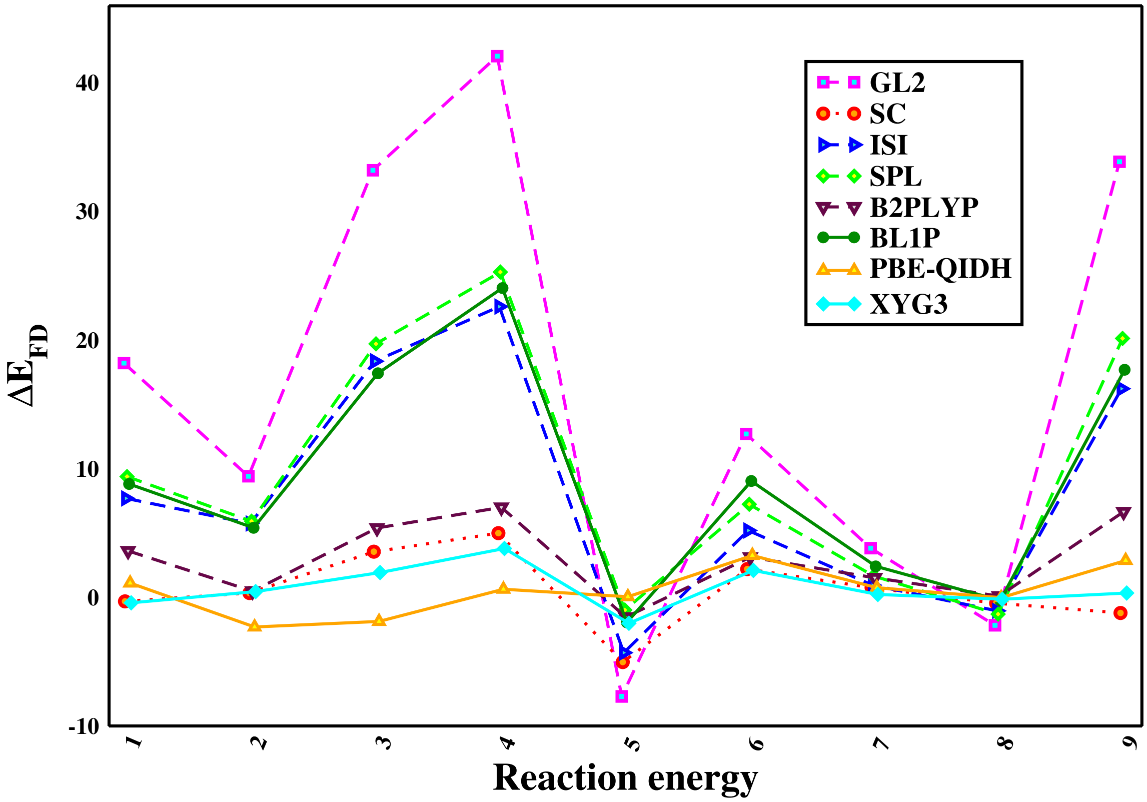

Figure S10: The individual functional driven errors ΔE_{FD} (in kcal/mol) for the reaction energy dataset. The numbering is described in the first column of Tab. S16.

Table S16: The closed-shell reaction energies for *ab initio* functionals (in kcal/mol) obtained for several trial orbitals, mean error (ME), mean absolute error (MAE), and mean absolute relative error (MARE) calculated with respect to CCSD(T) reference.

					@SCF		@PBE		@PBE0		WY[@HF]		WY[@MP2]		WY[@CCSD]		WY[@CCSD(T)]	
No.		CCSD(T)	CCSD	MP2	GL2[a]	SC	GL2	SC	GL2	SC	GL2	SC	GL2	SC	GL2	SC	GL2	SC
1	$H_2O_2+H_2 \rightarrow 2H_2O$	-86.74	-88.52	-91.27	–	-88.34	-69.88	-87.36	-79.38	-88.26	-76.86	-89.11	-69.05	-87.25	-69.85	-87.34	-68.52	-87.05
2	$CO + H_2 \rightarrow CH_2O$	-4.89	-5.05	-5.29	nc	-4.73	3.41	-5.01	-1.01	-5.00	0.73	-5.45	7.61	-3.86	3.66	-4.73	4.53	-4.54
3	$CO + 3H_2 \rightarrow CH_4 + H_2O$	-64.82	-66.39	-66.52	-16.63	-63.43	-32.03	-61.27	-48.53	-62.51	-48.44	-65.44	-28.37	-60.57	-34.72	-61.96	-31.61	-61.27
4	$N_2 + 3H_2 \rightarrow 2NH_3$	-38.83	-40.44	-38.34	14.30	-35.58	2.69	-34.19	-16.43	-35.05	-8.58	-36.73	5.69	-33.29	1.96	-34.13	3.25	-33.84
5	$BH_3 + 3HF \rightarrow BF_3 + 3H_2$	-90.74	-89.84	-91.43	-100.98	-93.83	-105.00	-98.50	-99.57	-96.73	-88.96	-92.61	-97.96	-95.59	-96.06	-95.09	-98.44	-95.77
6	$HCN + H_2O \rightarrow CO + NH_3$	-12.01	-12.69	-9.03	–	-9.88	0.83	-10.07	-3.73	-9.91	2.28	-9.27	-0.78	-10.12	1.72	-9.56	0.70	-9.81
7	$HCN + NH_3 \rightarrow N_2 + CH_4$	-37.99	-38.64	-37.22	–	-37.73	-33.89	-37.15	-35.83	-37.38	-37.58	-37.99	-34.84	-37.40	-34.97	-37.39	-34.17	-37.24
8	$2HF \rightarrow (HF)_2$	-2.97	-2.65	-2.76	-5.98	-3.18	-5.56	-3.72	-4.37	-3.48	-3.76	-3.08	-4.96	-3.36	-4.80	-3.34	-5.14	-3.42
9	$H_2 + F_2 \rightarrow 2HF$	-133.93	-136.73	-141.83	–	-136.49	-103.44	-136.16	-121.09	-137.32	-114.11	-138.19	-100.31	-135.31	-102.21	-135.54	-100.06	-135.14
	ME		-0.89	-1.20	22.01	-0.03	14.45	-0.06	7.00	-0.30	10.85	-0.55	16.66	0.68	15.30	0.42	15.94	0.54
	MAE		1.16	2.19	28.64	1.63	18.19	2.50	9.27	2.26	11.02	1.63	18.71	2.27	16.88	1.97	18.13	2.09
	MARE [%]		3.70	6.46	80.97	5.13	65.56	8.29	34.66	6.98	43.65	5.84	72.11	8.78	62.13	6.77	66.23	7.52

[a] SCF GL2 have not converged for CH_2O, F_2, H_2O_2, HCN systems. Thus, the ME, MAE, and MARE are calculated without these cases.

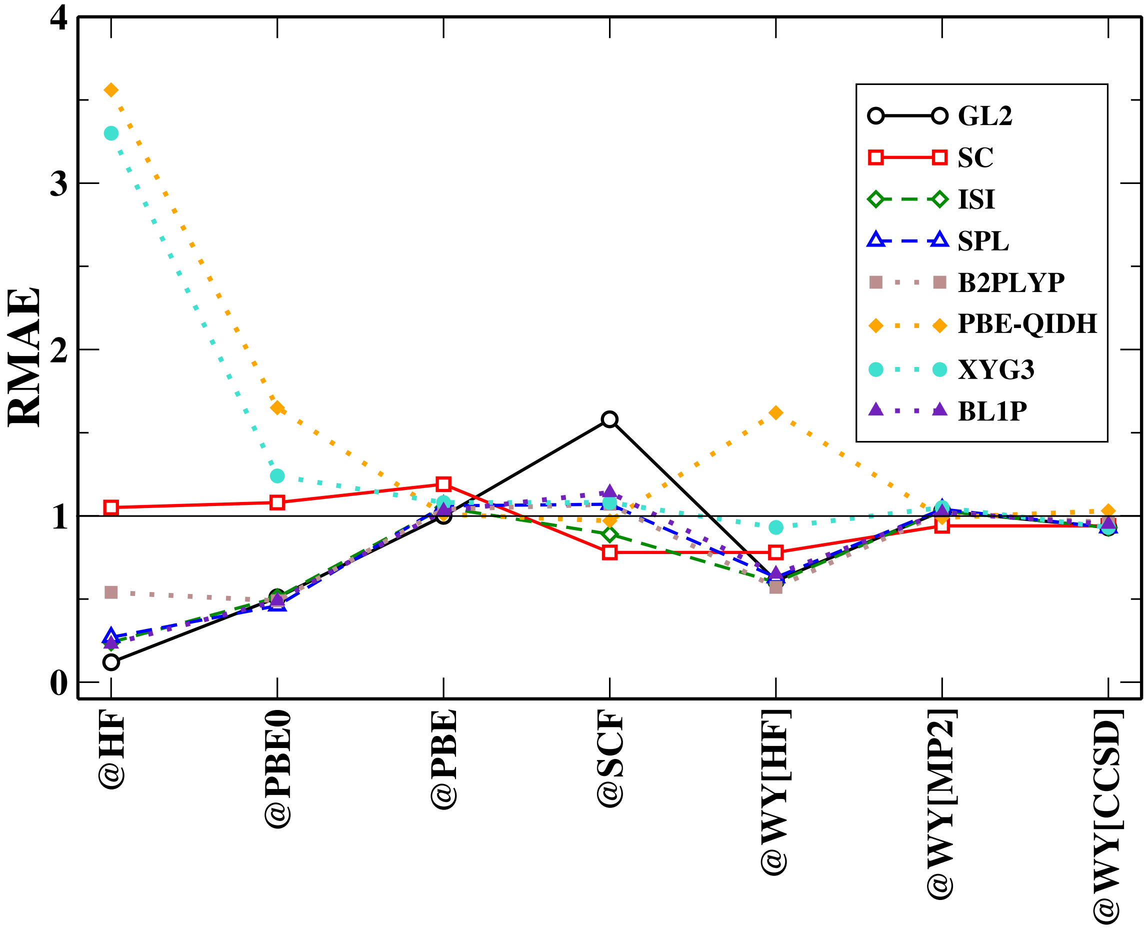

Figure S11: Relative mean absolute error (RMAE), for the reaction energies computed with different functionals and references. All calculations have been performed in an uncontracted aug-cc-pVTZ basis set.

Table S17: The closed-shell reaction energies for ISI functionals (in kcal/mol) obtained for several trial orbitals, mean error (ME), mean absolute error (MAE), and mean absolute relative error (MARE) calculated with respect to CCSD(T) reference.

						ISI			
	CCSD(T)	@HF	@PBE	PBE0	@SCF	@WY[HF]	@WY[MP2]	@WY[CCSD]	@WY[CCSD(T)]
H_2O_2+H_2 → $2H_2O$	-86.74	-92.06	-79.54	-84.55	-79.31	-83.64	-79.35	-79.69	-79.04
CO+H_2→CH_2O	-4.89	-5.08	0.24	-2.24	1.87	-1.33	2.46	0.39	0.86
CO+3H_2 →CH_4+H_2O	-64.82	-66.87	-45.74	-55.23	-45.98	-56.14	-44.77	-48.11	-46.46
N_2+3H_2→2NH_3	-38.83	-39.79	-16.56	-26.37	-15.47	-22.64	-15.08	-16.84	-16.23
BH_3+3HF→BF_3+3H_2	-90.74	-91.20	-101.87	-97.26	-93.65	-88.56	-94.93	-93.53	-95.04
HCN+H_2O→CO+NH3	-12.01	-11.32	-7.67	-8.97	-6.41	-5.32	-7.66	-6.16	-6.81
HCN+NH_3→N_2+CH_4	-37.99	-38.40	-36.85	-37.83	-36.92	-38.82	-37.36	-37.44	-37.04
2HF→$(HF)_2$	-2.97	-2.61	-4.86	-3.81	-3.71	-3.17	-3.91	-3.82	-4.01
H_2 + F_2→2HF	-133.93	-143.30	-119.79	-129.61	-140.28	-125.92	-117.93	-118.82	-117.70
ME		-1.97	6.69	3.00	5.89	5.26	8.26	7.65	7.94
MAE		2.20	9.59	4.64	8.12	5.49	9.41	8.46	9.12
MARE [%]		4.67	36.19	18.68	35.39	22.70	37.46	32.38	34.53

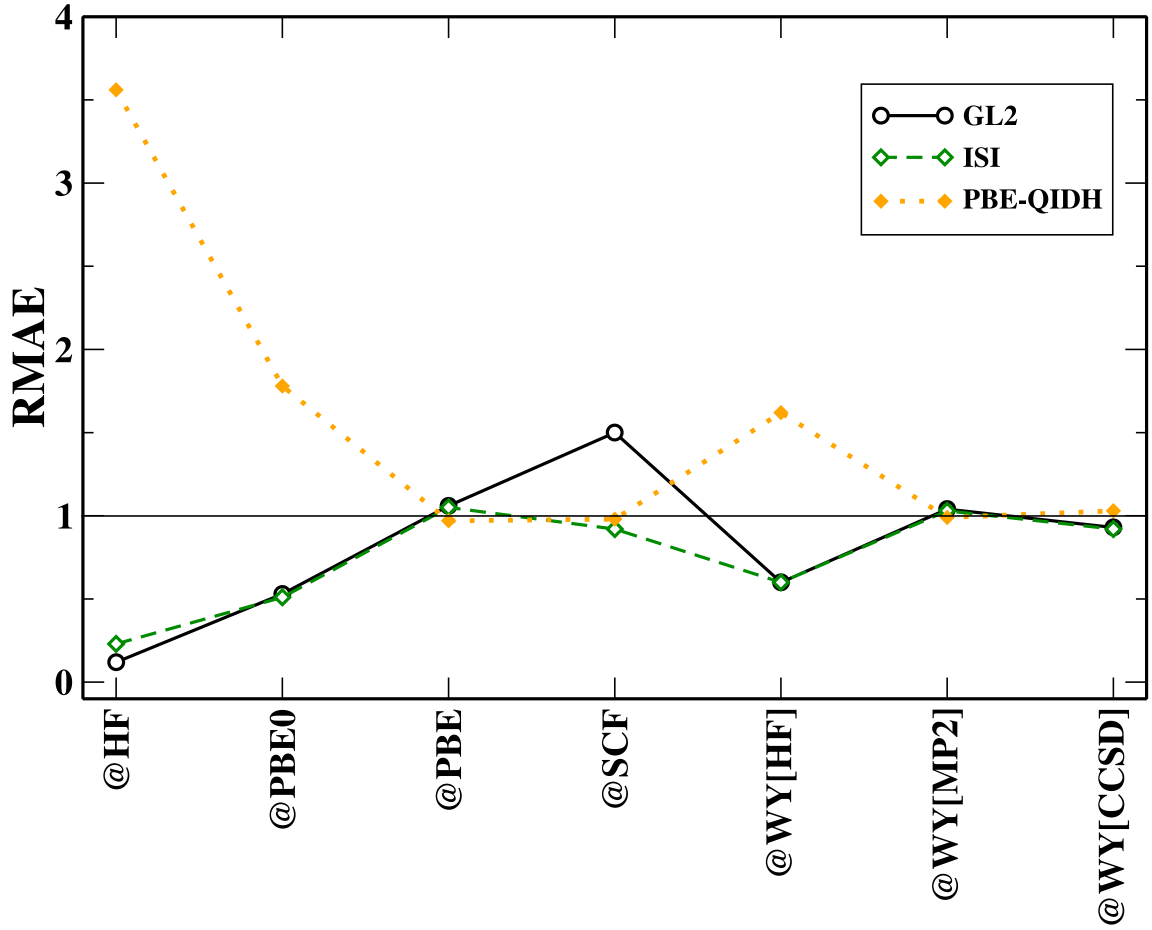

Figure S12: Relative mean absolute error (RMAE), for the reaction energies computed with different functionals and references. All calculations have been performed in an uncontracted aug-cc-pVQZ basis set.

Table S18: The closed-shell reaction energies for SPL functionals (in kcal/mol) obtained for several trial orbitals, mean error (ME), mean absolute error (MAE), and mean absolute relative error (MARE) calculated with respect to CCSD(T) reference.

		SPL							
	CCSD(T)	@HF	@PBE	@PBE0	@SCF	@WY[HF]	@WY[MP2]	@WY[CCSD]	@WY[CCSD(T)]
H_2O_2+H_2→2H_2O	-86.74	-92.54	-77.91	-84.21	-76.25	-83.09	-77.67	-78.21	-77.34
CO+H_2→CH_2O	-4.89	-5.56	0.21	-2.67	3.05	-1.72	3.15	0.42	1.03
CO+3H_2 →CH_4+H_2O	-64.82	-68.12	-44.08	-55.62	-42.10	-56.90	-42.83	-47.23	-45.10
N_2+3H_2 →2NH_3	-38.83	-40.85	-13.83	-26.06	-10.80	-21.62	-11.86	-14.39	-13.54
BH_3+3HF→BF_3+3H_2	-90.74	-89.32	-99.99	-94.95	-90.83	-84.60	-91.53	-89.96	-91.72
HCN+H_2O→CO+NH3	-12.01	-10.85	-5.79	-7.77	-4.18	-3.21	-5.83	-4.00	-4.77
HCN+NH_3→N_2+CH_4	-37.99	-38.12	-36.03	-37.33	-35.48	-38.49	-36.79	-36.84	-36.33
2HF→$(HF)_2$	-2.97	-2.61	-5.35	-3.99	-4.09	-3.27	-4.15	-4.03	-4.27
H_2 + F_2 → 2HF	-133.93	-143.69	-116.27	-128.38	-116.39	-123.93	-113.97	-115.26	-113.79
ME		-2.08	8.21	3.55	10.65	6.23	10.16	9.27	9.67
MAE		2.73	10.79	4.71	10.92	6.41	10.60	9.50	10.18
MARE [%]		6.87	41.26	19.50	44.93	24.94	43.13	36.52	39.13

Table S19: The closed-shell reaction energies for B2PLYP functionals (in kcal/mol) obtained for several trial orbitals, mean error (ME), mean absolute error (MAE), and mean absolute relative error (MARE) calculated with respect to CCSD(T) reference.

							DH			
	CCSD(T)	@HF	@PBE	@PBE0	@SCF	GKS	@WY[HF]	@WY[MP2]	@WY[CCSD]	@WY[CCSD(T)]
H_2O_2 + H_2→$2H_2O$	-86.74	-88.71	-83.15	-85.11	-83.19	-86.55	-85.13	-83.25	-83.25	-83.12
CO + H_2→CH_2O	-4.89	-6.82	-4.25	-5.77	-4.07	-6.60	-5.08	-4.23	-4.53	-4.41
CO + $3H_2$ → CH_4 + H_2O	-64.82	-67.89	-58.99	-62.87	-58.71	-65.48	-63.03	-59.22	-59.79	-59.42
N_2 + $3H_2$→ $2NH_3$	-38.83	-42.50	-31.63	-36.49	-31.29	-39.63	-34.50	-31.55	-32.00	-31.83
BH_3 + 3HF →BF_3 + $3H_2$	-90.74	-90.59	-92.46	-93.03	-92.35	-92.42	-89.67	-92.22	-91.94	-92.27
HCN + H_2O→ CO + NH_3	-12.01	-11.79	-8.93	-10.31	-8.98	-11.11	-8.69	-8.80	-8.78	-8.88
HCN + NH_3 → N_2 + CH_4	-37.99	-37.18	-36.29	-36.69	-36.40	-36.97	-37.22	-36.48	-36.57	-36.47
2HF→$(HF)_2$	-2.97	-2.48	-2.87	-2.99	-2.88	-2.87	-2.56	-2.84	-2.83	-2.87
H_2 + F_2 → 2HF	-133.93	-137.61	-127.27	-131.49	-126.89	-134.19	-130.53	-127.37	-127.50	-127.28
ME		-1.41	3.01	0.91	3.13	-0.32	1.83	2.99	2.86	2.93
MAE		1.78	3.39	1.62	3.49	0.81	1.88	3.32	3.12	3.27
MARE [%]		8.80	9.45	5.74	9.91	6.00	7.43	9.58	8.67	8.94

Table S20: The closed-shell reaction energies for PBE-QIDH functionals (in kcal/mol) obtained for several trial orbitals, mean error (ME), mean absolute error (MAE), and mean absolute relative error (MARE) calculated with respect to CCSD(T) reference.

							DH			
	CCSD(T)	@HF	@PBE	@PBE0	@SCF	GKS	@WY[HF]	@WY[MP2]	@WY[CCSD]	@WY[CCSD(T)]
H_2O_2+H_2 →$2H_2O$	-86.74	-91.65	-85.48	-87.88	-85.65	-90.18	-87.74	-85.60	-85.75	-85.62
CO+H_2→CH_2O	-4.89	-10.46	-6.96	-8.90	-6.79	-10.27	-8.15	-7.01	-7.34	-7.19
CO+$3H_2$→CH_4+H_2O	-64.82	-76.82	-66.22	-71.11	-65.91	-75.11	-70.85	-66.45	-67.09	-66.69
N_2+$3H_2$→$2NH_3$	-38.83	-51.30	-37.87	-44.04	-37.62	-49.13	-41.54	-37.92	-38.40	-38.20
BH_3+3HF→BF_3+$3H_2$	-90.74	-89.37	-90.69	-91.24	-90.76	-90.85	-87.92	-90.82	-90.35	-90.68
HCN+H_2→CO+NH3	-12.01	-12.31	-8.85	-10.51	-8.98	-11.97	-8.40	-8.79	-8.62	-8.75
HCN+NH_3→N_2+CH_4	-37.99	-37.83	-37.20	-37.58	-37.27	-37.95	-37.70	-37.31	-37.31	-37.24
2HF→$(HF)_2$	-2.97	-2.75	-3.00	-3.14	-2.98	-3.04	-2.69	-3.01	-2.96	-2.99
H_2 + F_2→2HF	-133.93	-142.80	-130.68	-135.92	-130.53	-140.35	-134.83	-130.98	-131.33	-131.06
ME		-4.71	0.66	-1.93	0.71	-3.99	-0.77	0.56	0.42	0.50
MAE		5.10	1.44	2.36	1.39	4.01	2.32	1.42	1.47	1.43
MARE [%]		20.99	8.93	14.22	8.36	18.26	14.26	9.09	9.84	9.45

Table S21: The closed-shell reaction energies for XYG3 functionals (in kcal/mol) obtained for several trial orbitals, mean error (ME), mean absolute error (MAE), and mean absolute relative error (MARE) calculated with respect to CCSD(T) reference.

							DH			
	CCSD(T)	@HF	@PBE	@PBE0	@SCF	GKS	@WY[HF]	@WY[MP2]	@WY[CCSD]	@WY[CCSD(T)]
H_2O_2+H_2 →$2H_2O$	-86.74	-92.15	-87.03	-89.11	-87.27	-88.73	-88.79	-87.16	-87.22	-87.16
CO+H_2→CH_2O	-4.89	-7.72	-4.01	-5.98	-4.11	-5.65	-5.35	-4.36	-4.57	-4.44
CO+$3H_2$→CH_4+H_2O	-64.82	-71.88	-62.33	-66.82	-62.42	-66.10	-66.11	-62.77	-63.16	-62.89
N_2+$3H_2$ →$2NH_3$	-38.83	-47.04	-34.63	-40.44	-34.61	-39.36	-37.67	-34.84	-35.18	-35.03
BH_3+3HF→BF_3+$3H_2$	-90.74	-92.77	-91.88	-93.14	-92.35	-92.98	-91.13	-92.78	-92.65	-92.75
HCN+H_2O→CO+NH3	-12.01	-13.66	-9.92	-11.59	-10.02	-11.28	-9.81	-9.84	-9.86	-9.91
HCN+NH_3 →N_2+CH_4	-37.99	-38.50	-37.62	-37.97	-37.83	-38.02	-38.25	-37.77	-37.83	-37.77
2HF→$(HF)_2$	-2.97	-3.15	-3.00	-3.29	-3.11	-3.27	-2.94	-3.11	-3.11	-3.11
H_2 + F_2→ 2HF	-133.93	-143.71	-133.07	-137.87	-133.44	-137.05	-136.64	-133.55	-133.77	-133.60
ME		-4.19	1.05	-1.48	0.86	-1.06	-0.42	0.75	0.62	0.69
MAE		4.19	1.37	1.58	1.37	1.22	1.17	1.33	1.18	1.27
MARE [%]		14.12	6.01	5.81	6.07	4.68	4.36	5.62	4.90	5.31

Table S22: The closed-shell reaction energies for BL1P functionals (in kcal/mol) obtained for several trial orbitals, mean error (ME), mean absolute error (MAE), and mean absolute relative error (MARE) calculated with respect to CCSD(T) reference.

							DH			
	CCSD(T)	@HF	@PBE	@PBE0	@SCF[a]	GKS	@WY[HF]	@WY[MP2]	@WY[CCSD]	@WY[CCSD(T)]
H_2O_2 + H_2 → $2H_2O$	-86.74	-90.55	-78.51	-83.92	-75.48	-89.30	-82.26	-78.08	-78.42	-77.92
CO + H_2→CH_2O	-4.89	-6.14	0.58	-2.97	2.92	-5.88	-1.61	1.35	0.14	0.52
CO + $3H_2$ →CH_4 + H_2O	-64.82	-67.73	-46.42	-56.61	-41.08	-66.26	-55.63	-46.46	-48.51	-47.39
N_2 + $3H_2$→$2NH_3$	-38.83	-40.90	-14.60	-27.04	-10.17	-38.97	-21.10	-13.84	-15.32	-14.78
BH_3 + 3HF→ BF_3 + $3H_2$	-90.74	-91.33	-95.35	-95.08	-94.09	-92.06	-88.68	-92.51	-92.00	-92.68
HCN + H_2O → CO + NH_3	-12.01	-10.53	-3.00	-6.32	-2.87	-9.92	-2.69	-3.12	-2.69	-2.96
HCN + NH_3 →N_2 + CH_4	-37.99	-37.36	-34.81	-35.88	-33.77	-37.20	-37.22	-35.75	-35.88	-35.58
2HF → $(HF)_2$	-2.97	-2.73	-3.42	-3.37	-3.32	-2.91	-2.77	-3.13	-3.13	-3.21
H_2 + F_2 → 2HF	-133.93	-140.70	-117.56	-128.68	–	-138.59	-124.07	-116.25	-117.16	-116.24
ME		-1.67	8.87	3.67	10.14	-0.91	6.32	9.46	8.88	9.18
MAE		2.20	9.99	4.72	11.07	1.56	6.32	9.89	9.20	9.67
MARE [%]		7.52	36.45	17.82	48.24	5.83	25.33	36.76	33.38	34.97

[a] SCF calculations have not converged for F_2. Thus, the ME, MAE, and MARE are calculated without this case.
